# Isotope Calculation Gadgets: A Series of Software for Isotope-Tracing Experiments in Garuda Platform

**DOI:** 10.3390/metabo12070646

**Published:** 2022-07-14

**Authors:** Nobuyuki Okahashi, Yuki Yamada, Junko Iida, Fumio Matsuda

**Affiliations:** 1 Department of Bioinformatic Engineering, Graduate School of Information Science and Technology, Osaka University, 1-5 Yamadaoka, Suita 565-0871, Japan; n-okahashi@ist.osaka-u.ac.jp (N.O.); yuki.yamada@ist.osaka-u.ac.jp (Y.Y.); 2 Department of Biotechnology, Osaka University Shimadzu Analytical Innovation Research Laboratory, Graduate School of Engineering, Osaka University, 2-1 Yamadaoka, Suita 565-0871, Japan; ji@shimadzu.co.jp; 3 Industrial Biotechnology Initiative Division, Institute for Open and Transdisciplinary Research Initiatives, Osaka University, 2-1 Yamadaoka, Suita 565-0871, Japan; 4 Analytical and Measuring Instruments Division, Shimadzu Corporation, 1 Nishinokyo Kuwabara-cho, Nakagyo-ku, Kyoto 604-8511, Japan

**Keywords:** isotope tracing, mass spectrometry, isotope correction, flux ratio analysis, Garuda

## Abstract

Isotope tracing is a powerful technique for elucidating intracellular metabolism. Experiments utilizing this technique involve various processes, such as the correction of natural isotopes. Although some previously developed software are available for these procedures, there are still time-consuming steps in isotope tracing including the creation of an isotope measurement method in mass spectrometry (MS) and the interpretation of obtained labeling data. Additionally, these multi-step tasks often require data format conversion, which is also time-consuming. In this study, the Isotope Calculation Gadgets, a series of software that supports an entire workflow of isotope-tracing experiments, was developed in the Garuda platform, an open community. Garuda is a graphical user interface-based platform that allows individual operations to be sequentially performed, without data format conversion, which significantly reduces the required time and effort. The developed software includes new features that construct channels for isotopomer measurements, as well as conventional functions such as natural isotope correction, the calculation of fractional labeling and split ratio, and data mapping, thus facilitating an overall workflow of isotope-tracing experiments through smooth functional integration.

## 1. Introduction

Isotope tracing has become an indispensable technique for studying the metabolism in various systems ranging from microorganisms to mammalian cells [1,2,3]. Generally, this technique is performed by feeding stable isotope-labeled substrates to cells of interest and analyzing a series of metabolites containing different numbers of the isotope (isotopologue) using mass spectrometry (MS). Isotope tracing requires several special operations not found in metabolomics [1,2]; thus, various software packages have been developed to perform these processes. The most important task is to correct the effects of natural isotopes [1,2]. The obtained raw labeling data include naturally occurring isotopes such as ^13^C (1.07%), ^2^H (0.01%), ^17^O (0.04%), ^18^O (0.21%), and ^15^N (0.36%), which leads to a bias in tracer-derived labeling data [4]. In particular, the effect is significant in widely used gas chromatography mass spectrometry (GC–MS) analysis because ^29^Si (4.69%) and ^30^Si (3.09%) are relatively abundant in the silyl groups introduced into metabolites by derivatization [4]. In liquid chromatography (LC)–MS analysis, the bias is non-negligible, especially for molecules with larger molecular weights such as lipids. Various software programs for correcting raw data using mathematical calculations are available and widely used [5,6,7,8,9,10,11]. In recent years, various functions have been added to conventional natural isotope correction. For example, ICT, IsoCorrectoR and IsoCor v2 can correct the effects of natural isotopes for tandem mass spectrometry data [6,8,9]. PyNac, IsoCorrectoR, IsoCor v2, and PolyMID support the correction of high-resolution mass spectrometry data [5,8,9,11]. The effect of tracer impurity can be considered by ICT, IsoCorrectoR, IsoCor v2, and PolyMID [6,8,9,11]. IsoCorrectoR and MIDcor can compute theoretical labeling data to compare them to the measured data or deconvolute the overlapped peaks [7,8]. Escher-Trace can visualize corrected data on metabolic pathway maps [10].

However, there are still time-consuming steps in isotope tracing. Corrected labeling data are not always suitable for inferring metabolic states and must often be converted to other forms, such as fractional labeling and flux split ratio [1]. For example, the contribution of a uniformly labeled substrate to intracellular metabolism can be evaluated by calculating the fractional labeling accumulated in pathway intermediates, revealing the dependence of cells on a particular substrate for specific biological processes [1,2]. Flux ratio analysis provides a split ratio at the metabolic branch, such as for glycolysis and the pentose phosphate (PP) pathway, allowing for the quantitative understanding of the importance of a particular metabolic pathway [1,12,13,14,15].

Another task that should be addressed is the creation of complex mass-to-charge (*m*/*z*) lists for accurate labeling measurements [16]. *m*/*z* lists can be used to develop selected ion monitoring methods or extract ion chromatograms from scan data in mass spectrometry. The creation of these lists is located in the first step and is often a critical barrier to start isotope tracing. For example, in the case of ^13^C-tracers, it is necessary to create as many channels as the number of carbon atoms in the backbone of the target metabolites. In addition, it should be noted that the extra channels of natural isotopes must be prepared for uniformly labeled metabolites to properly correct the effect of natural isotopes [16]. This is often overlooked by beginners and needs to be appropriately guided for accurate isotope measurements. Channel creation for the multiple reaction monitoring (MRM) mode is much more complicated because the combinations of isotope numbers in the precursor and product ions are enormous [17,18,19,20]. These complex procedures have not been supported by publicly available software.

The multiple aforementioned steps, including method development, natural isotope correction, data interpretation, and data mapping, require several calculations and data conversion that result in cumbersome and time-consuming isotope-tracing experiments. To address these issues, we developed a series of open-source software “Isotope Calculation Gadgets” in Garuda, an open, community-driven, common platform that provides a framework for connecting, discovering, and navigating through different applications, databases, and services in biology and medicine [21]. In the Garuda platform, the analysis is performed using “gadget” software that is divided into functional units to perform particular operations. A gadget can be connected to another by researchers to smoothly transfer data, which enables seamless data processing. The Garuda community accepts software developed by third parties and can work with existing gadgets of MS data processing and mapping (http://www.garuda-alliance.org/gadgetpack/shimadzu/ (accessed on 1 June 2022)). The presented software covers an overall workflow of isotope-tracing experiments, such as method development for isotopomer channels and data interpretation, as well as the correction of natural isotopes and data visualization on a metabolic map in a platform. Users can freely arrange a data processing workflow by combining different software depending on their research objectives in a graphical user interface without time-consuming data format conversion.

## 2. Results

### 2.1. Overview of This Study

**Figure 1:**
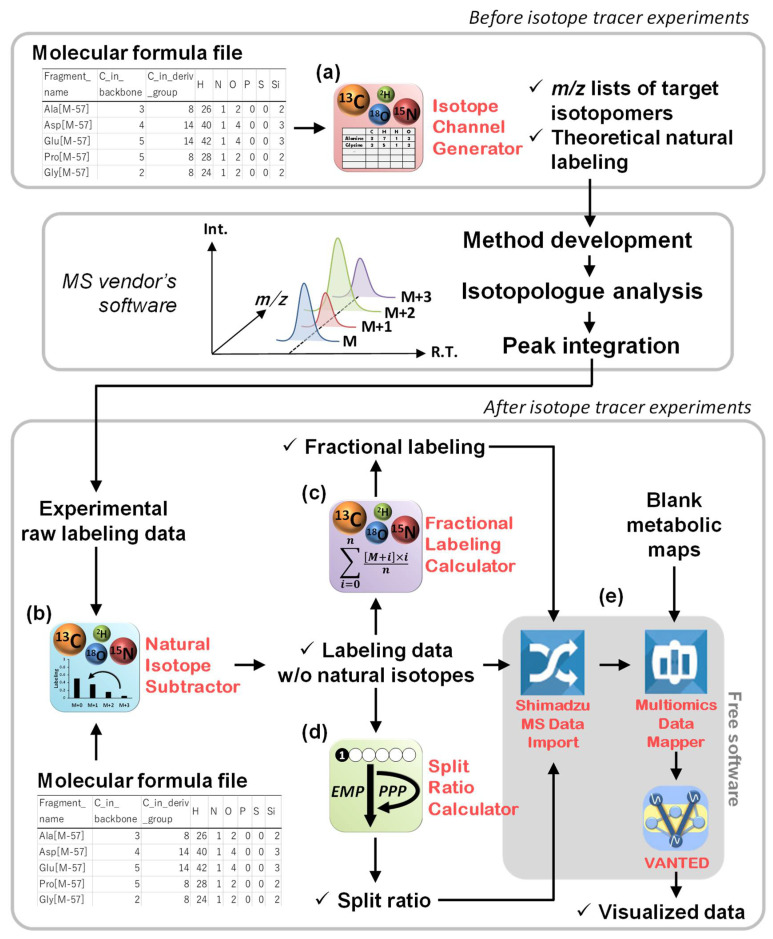
Overview of the Isotope Calculation Gadgets. The workflows of the (**a**) isotope channel generator, (**b**) natural isotope subtractor, (**c**) fractional labeling calculator, (**d**) split ratio calculator, and (**e**) free software for data mapping are illustrated.

In general isotope-tracing experiments, cells are cultured with substrates containing isotopes such as ^13^C or ^15^N and the isotopologue abundance of metabolites extracted from cells is measured by mass spectrometry to investigate the intracellular metabolic flow. We developed four gadgets supporting isotope-tracing experiments written in Python ver. 3.7.6 and operated by a graphical user interface in Garuda platform ver.1.4. A tutorial can be found at https://nobuyukiokahashi.github.io/github.io-Isotope_calc_gadgets_tutorial/ (accessed on 1 June 2022). The workflow of the Isotope Calculation Gadgets is divided into two major parts: before and after an isotope-tracing experiment (Figure 1). The former part is assisted by the creation of methods for labeling analysis (Section 2.2 and Figure 1a), and the latter is supported by data processing, including the correction of natural isotopes (Section 2.3 and Figure 1b) and the calculation of fractional labeling and split ratio (Section 2.4 and Section 2.5 and Figure 1c,d). Mapping labeling data on a metabolic map can be performed using cooperative software freely available in Garuda, independent of MS manufacturer (Section 2.6, Figure 1e). The utility of this tool was demonstrated in ^13^C-labeling analyses using GC–MS and LC-QTOF/MS. Two colorectal cancer cell lines, HCT116 and WiDr, were cultured with ^13^C-labeled glucose and glutamine, followed by the measurement of the intermediate metabolites in central carbon metabolism by GC–MS (Section 2.7). Furthermore, budding yeast, *Saccharomyces cerevisiae* BY4947, was cultured with [U-^13^C]glucose, and then lipids were analyzed with LC-QTOF/MS (Section 2.8). The conditions of the culture experiments are described in detail in Section 3.

### 2.2. Channel Creation by the Isotope Channel Generator

To develop an MS channel for monitoring isotopologues, the isotope channel generator creates an *m*/*z* list of metabolites to be measured by entering molecular formulas of target fragment ions, a tracer atom, and the number of additional channels for natural isotopes (Figure 1a). The *m*/*z* list can be used to create extract ion chromatograms from scan data and target channels of the selected ion monitoring (SIM) mode. The gadget can also generate an *m*/*z* list of precursor and product ions for an MRM mode from the molecular formula of the precursor and product ions. To experimentally validate the accuracy of the created channels, the isotope channel generator also computes the natural labeling ratio of target metabolites based on Equation (1):

(1) $$MDV_{raw}=CM\times MDV_{corr}$$

where $MDV_{raw}$ and $MDV_{corr}$ are raw and corrected (i.e., with and without natural isotopes, respectively) mass isotopologue distribution vector (MDV), a vector with isotopologue abundance ratios whose sum equals one, and CM is a correction matrix composed of the natural isotope ratio of each atom [22]. Since the labeling of chemical standards and cellular extracts in a natural environment is consistent with theoretical natural isotope abundance ratios, comparing the analytical results of the developed method with the theoretical values allows for the selection of fragments with accurate labeling and the optimization of chromatographic conditions to separate foreign peaks.

### 2.3. Correction of the Effects of Naturally Occurring Isotopes by the Natural Isotope Subtractor

After a tracing-culture experiment and subsequent labeled metabolite analysis, the effect of natural isotopes is corrected from raw MS data. The natural isotope subtractor converts the area of the obtained isotopologue peaks into $MDV_{raw}$, and $MDV_{corr}$ is computed by multiplying the inverse matrix of CM ($CM^{-1}$), as in Equation (2) [22]:

(2) $$MDV_{corr}=CM^{-1}\times MDV_{raw}$$

Users are free to choose the number and types of natural isotope elements they want to correct because this software supports low-resolution data, which may contain overlaps of multiple element-derived isotopes. The purity of the administered tracer can be corrected using the following Equation (3):

(3) $$MDV_{labeled}=\frac{MDV_{meas}-\left(1-\alpha \right)\cdot MDV_{natural}}{\alpha }$$

where α is the tracer purity and $MDV_{meas},$ $MDV_{labeled},$ and $MDV_{natural}$ are MDVs of metabolites actually measured, truly labeled by tracer, and found in nature, respectively. This correction is essential in systems where it is not possible to replace all originally present natural substrates with tracers. The corrected labeling data can be used for data interpretation via further data conversion.

### 2.4. Data Interpretation Using the Fractional Labeling Calculator

Calculating fractional labeling can determine the percentage of a particular substrate-derived atom that reaches specific metabolites, that is, the dependence on that substrate for the biosynthesis of metabolites. Fractional labeling is given by the following Equation (4) [1]:

(4) $$\mathrm{Fractional}\;\mathrm{labeling}=\overset{n}{\underset{i=0}{\sum }}\frac{\left[M+i\right]\times i}{n}$$

where *n* is the maximal number of elements in the metabolites of interest and [M + i] is the ratio of the *i*th element of the corrected MDV. Because *n* and the number of [M + i] elements are dependent on each metabolite, it is time-consuming to calculate them manually using general spreadsheet software when several metabolites are simultaneously measured. The fractional labeling calculator takes the data corrected by the natural isotope subtractor and calculates the fractional labeling for each measured fragment within a few seconds.

### 2.5. Data Interpretation Using the Split Ratio Calculator

**Figure 2:**
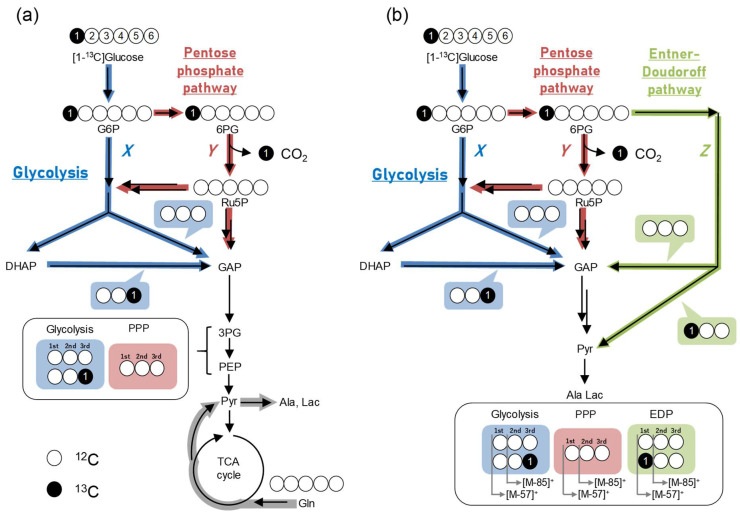
Carbon atom transitions of glucose metabolism. Metabolic pathways in (**a**) mammalian and (**b**) bacterial cells. White and black circles represent ^12^C and ^13^C, respectively, in the metabolite backbone. Double arrows indicate omitted multiple reactions.

The split ratio of fluxes in a branching metabolic network provides key information regarding the importance of the pathway in a biological process. Glycolysis (the Embden–Meyerhof–Parnas (EMP) pathway) and the PP pathway are important intracellular branching pathways. The flux ratio flowing into each pathway can be resolved by a tracing experiment using [1-^13^C]glucose [23]. This analysis is based on the biochemical property that the number and location of tracer-derived ^13^C in downstream metabolites change depending on the pathway (Figure 2). As [1-^13^C]glucose is broken down into two molecules of 3-phosphoglycerate (3PG) and phosphoenolpyruvate (PEP) via glycolysis, the isotopomer ratio of M+0 (unlabeled) to M+1 (singly ^13^C-labeled) in downstream metabolites is 1:1, whereas the carbon at the first position is lost as CO_2_ and produces 5/3 molecules of unlabeled downstream metabolites when it is metabolized via the PP pathway (Figure 2a). This difference can be discerned by measuring the ^13^C-labeling of 3PG and PEP. The balance of labeled metabolites can be described using Equations (5) and (6):

(5) X+Y=1

(6) $$\left[M+0]:[M+1\right]=X+\frac{5}{3}Y:X$$

where *X* and *Y* are the split ratios of glycolysis and the PP pathway, respectively, and [M + 0] and [M + 1] are the M+0 and M+1 ratios of 3PG and PEP, respectively. The fragment ions of 3PG and PEP must contain all the skeletal carbons, which correspond to the [M-57]^+^ fragment in *tert*-butyldimethylsilyl (TBDMS) derivatization. The use of ^13^C-labeling of pyruvate, alanine, and lactate for the split ratio analysis of mammalian cells is not recommended because unlabeled glutamine-derived ^12^C will dilute the labeling (Figure 2a).

The Entner–Doudoroff (ED) pathway, another branch of glycolysis specifically observed in some bacteria, breaks down [1-^13^C]glucose into two molecules of downstream metabolites whose ^13^C positions are different from those produced via glycolysis (Figure 2b). The split ratio can be determined by measuring the ^13^C-labeling of two fragment ions containing the first-to-third and second-to-third skeletal carbon atoms (corresponding to [M-57]^+^ and [M-85]^+^ fragments, respectively), following Equations (7)–(9):

(7) X+Y+Z=1

(8) $$[M+0{]}_{123}:[M+1{]}_{123}=X+\frac{5}{3}Y+2Z:X\;$$

(9) $$[M+0{]}_{23}:[M+1{]}_{23}=X+\frac{5}{3}Y+Z:X+Z$$

where *X*, *Y*, and *Z* are the split ratios of glycolysis, the PP pathway, and the ED pathway, respectively; ${\left[M+0\right]}_{123}$ and ${\left[M+0\right]}_{23}$ correspond to the M + 0 ratio of downstream metabolites of fragment ions containing the first-to-third and second-to-third carbons in the backbone, respectively; and ${\left[M+1\right]}_{123}$ and ${\left[M+1\right]}_{23}$ correspond to the M + 1 ratio of the downstream metabolites of fragment ions containing the first-to-third and second-to-third carbons in the backbone, respectively. The ^13^C-labeling of alanine [M-57]^+^ and [M-85]^+^ measured by GC–MS is often used because it is relatively easy to obtain two fragments [23,24] (Figure 2b). In summary, this software enables the quick calculation of flux ratios in important branching pathways of glucose metabolism.

### 2.6. Data Mapping

The visualization of labeling data on a metabolic map is useful for obtaining insights into intracellular metabolism. The type of metabolic map that is suitable largely depends on the nature of the experiment, so there needs to be flexibility for individual researchers to optimize the procedure. Therefore, the Isotope Calculation Gadgets are designed to be connected to the data visualization software Visualization and Analysis of Networks conTaining Experimental Data (VANTED) [25], via a previously developed free software, Shimadzu MS data import and multiomics data mapper (http://www.garuda-alliance.org/gadgetpack/shimadzu/ (accessed on 1 June 2022)), which are compatible with any vendor’s MS. This pipeline enables seamless data transfer for visualization on a template map without manual data conversion. Ready-to-use blank maps for visualizing isotope labeling, fractional labeling, and split ratio are available in https://github.com/NobuyukiOkahashi/Isotope_gadgets (accessed on 1 June 2022), and they can be freely modified as the experimenter wishes. 

### 2.7. Example of Analysis Using GC–MS

To demonstrate the usefulness of the Isotope Calculation Gadgets, ^13^C-labeling experiments using two colorectal cancer cell lines, HCT116 and WiDr, were performed. The performance test of data processing was carried out on a Windows 10 Education 64 bit-based machine with Intel (R) Core^TM^ i5-8350U CPU @ 1.70 GHz and 16 GB random access memory. HCT116 and WiDr cells were cultured in a medium containing either unlabeled glucose and glutamine, [1-^13^C]glucose and unlabeled glutamine, [U-^13^C]glucose and unlabeled glutamine, or unlabeled glucose and [U-^13^C]glutamine in triplicate for 24 h, as previously described [26]. Extracted intracellular metabolites were analyzed using GC–MS via methoxyamination and TBDMS derivatization, as previously described [27].

**Figure 3:**
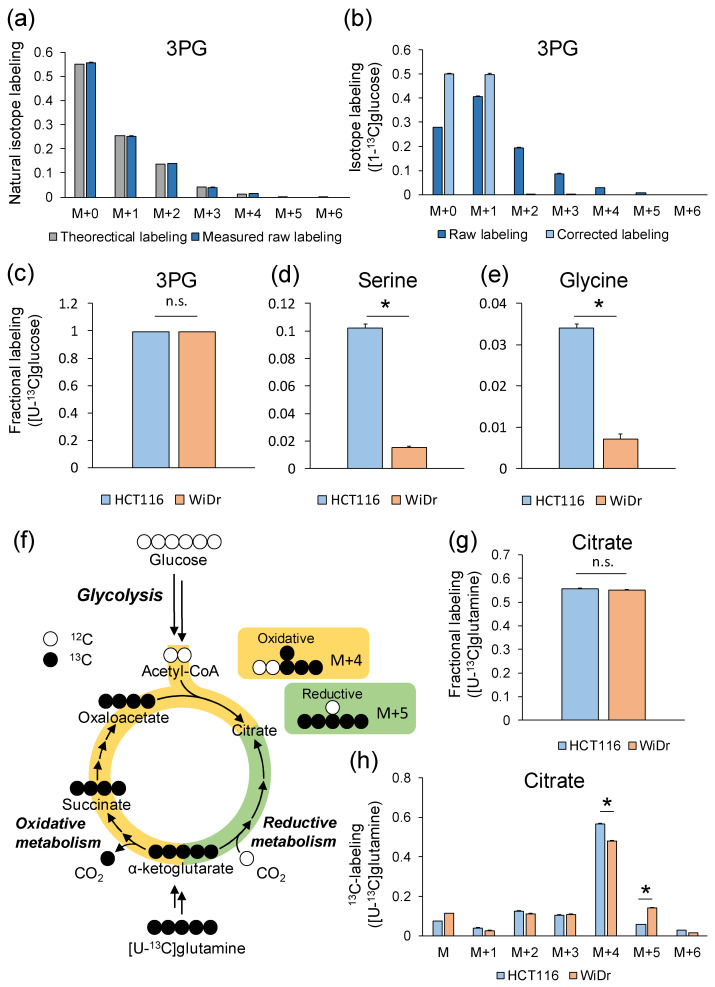
^13^C-labeling of intracellular metabolites in HCT116 and WiDr. (**a**) Theoretical and measured raw labeling of 3-phosphoglyceric acid (3PG) obtained from HCT116 cells cultured in unlabeled medium. (**b**) Raw and corrected labeling of 3PG obtained from HCT116 cells cultured in [1-^13^C]glucose. (**c**–**e**) Fractional labeling of (**c**) 3PG, (**d**) serine, and (**e**) glycine in HCT116 and WiDr cells cultured in [U-^13^C]glucose. (**f**) Atom mapping of oxidative (yellow) and reductive (green) glutamine metabolism. (**g**,**h**) (**g**) Fractional labeling and (**h**) corrected ^13^C-labeling of citrate in HCT116 and WiDr cells cultured in [U-^13^C]glutamine. Data are presented as the mean ± standard deviation (*n* = 3). *, *p* < 0.05 in Student’s *t*-test. n.s., no significance.

For GC–MS analysis, the isotope channel generator created a total of 117 GC–MS selected ion monitoring (SIM) channels, including three additional natural isotope channels per metabolite for 15 methoxyaminated and TBDMS-derivatized amino acids (alanine, serine, glycine, aspartate, glutamate, and proline) and intermediate metabolites (3PG, PEP, pyruvate, lactate, citrate, α-ketoglutarate, succinate, fumarate, and malate) in 2 s from a chemical formula file (Supplementary File S1). The measured raw labeling of derivatized 3PG obtained from HCT116 cells cultured in an unlabeled medium agreed well with the theoretical labeling (Figure 3a), indicating the correctness of the measurements.

A total of 336 row labeling data (2 cell lines × 4 tracer conditions × 14 detected metabolites × 3 repetitions; Supplementary File S2) were processed to correct the effect of natural isotopes by the natural isotope subtractor in 11 s. The isotopic steady state at the time point was confirmed in a previous study [27]. The calculations of fractional labeling and split ratio were finished within a second. All data were visualized on a metabolic map (Figures S1–S3).

In the experiment using [1-^13^C]glucose, the labeling pattern of 3PG should be M+0 and M+1, as shown in atom mapping (Figure 2a). However, the mass isotopologues of 3PG in HCT116 cells cultured in [1-^13^C]glucose were distributed from M+0 to M+5 (Figure 3b). This was due to the presence of natural isotopes, due to which parts of M+0 or M+1 were detected as M+2, M+3, M+4, and M+5. Correcting the effect of those natural isotopes converted M+2, M+3, M+4, and M+5 back to M+0 and M+1. As expected, the corrected labeling computed by the natural isotope subtractor showed 50.3% of M+0, 49.5% of M+1, and a slight ratio of other isotopologues (Figure 3b), implying a high glycolytic metabolism (Figure 2a). The fractional labeling of 3PG did not show significant differences in HCT116 and WiDr cultured in [U-^13^C]glucose (Figure 3c); however, those of serine and glycine synthesized from 3PG were significantly larger in HCT116 than in WiDr (Figure 3d,e), suggesting that HCT116 more actively synthesized serine and glycine from glucose than WiDr. The flux split ratio of glycolysis based on 3PG labeling was 99.9% and 97.1% in HCT116 and WiDr, respectively, and the flux to the PP pathway was nearly zero, indicating that both cell lines showed the Warburg effect, which is a hallmark of cancer cell metabolism. 

The M+4 and M+5 labeling of citrate obtained from [U-^13^C]glutamine was a marker of oxidative and glutamine metabolism [28] (Figure 3f). The effect of natural isotopes in citrate was corrected by the natural isotope subtracter based on Equation (2). Although the fractional labeling of citrate in HCT116 and WiDr cells cultured in [U-^13^C]glutamine was not significantly different (Figure 3g), the labeling of M+4 citrate in HCT-116 (56.7%) was higher than that of WiDr (53.6%), whereas that of M+5 citrate showed the opposite trend (5.9% and 8.8%, respectively, Figure 3h). This labeling signature indicated that the contribution level of glutamine to citrate biosynthesis is similar, whereas the biosynthetic pathway (namely the dependency of oxidative and reductive metabolism in the tricarboxylic acid cycle (TCA) cycle) differed between the HCT116 and WiDr cell lines. 

### 2.8. Example of Analysis Using LC–MS

**Figure 4:**
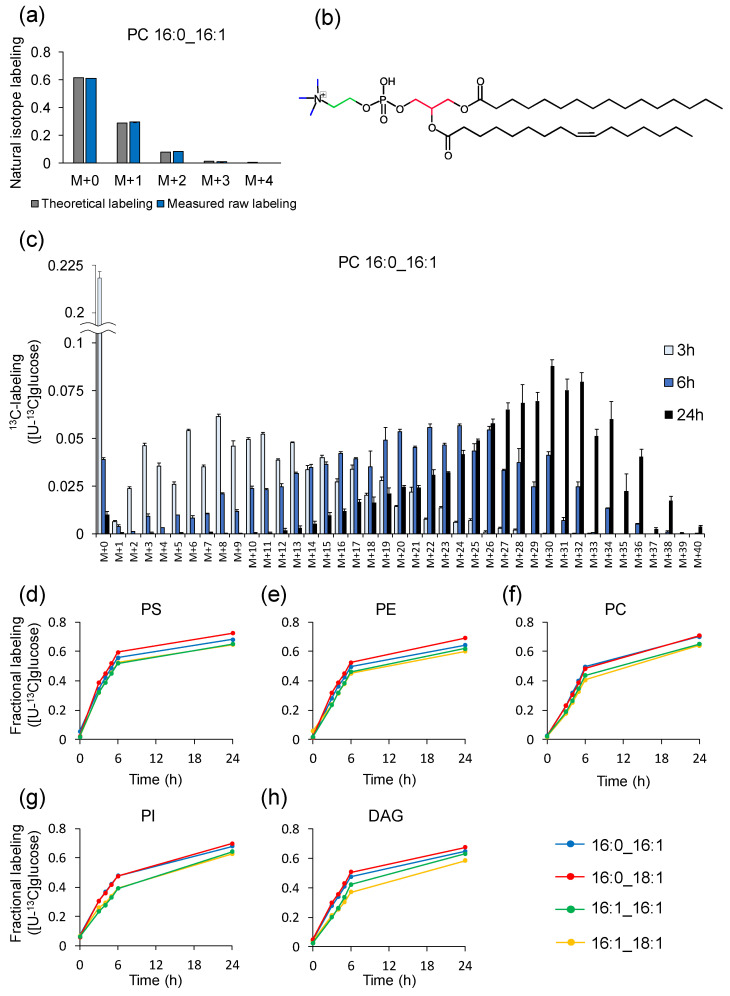
^13^C-labeling of glycerolipids in *S. cerevisiae* BY4947. (**a**) Theoretical and measured raw labeling of PC 16:0_16:1 obtained from *S. cerevisiae* cells cultured in unlabeled medium. (**b**) Chemical structure of 16:0_16:1. Carbons colored in red, blue, and green represent glycerol backbone, *N*-methyl group, and choline backbone, respectively. (**c**) Time-course of isotopologue distribution of PC 16:0_16:1 obtained from *S. cerevisiae* cells cultured in [U-^13^C]glucose medium. (**d**–**h**) Fractional labeling for (**d**) PS, (**e**) PE, (**f**) PC, (**g**) PI, and (**h**) DAG with (blue) 16:0_16:1, (red) 16:0_18:1, (green) 16:1_16:1, and (yellow) 16:1_18:1 acyl chain combinations. Data are presented as the mean ± standard deviation (*n* = 3).

The utility of Isotope Calculation Gadgets in LC–MS was also examined in a time-course analysis of ^13^C-labeling of glycerolipids in budding yeast. The *Saccharomyces cerevisiae* BY4947 strain was cultured in a synthetic dextrose (SD) medium containing either natural or [U-^13^C]glucose in triplicate. Whole yeast lipids were extracted with a single-phase extraction method [29] and analyzed with LC-QTOF/MS in a data-dependent acquisition mode (see Section 3). In total, 442 lipid molecular species were identified in positive and negative modes from the unlabeled samples by using MS-DIAL software [30], after which ^13^C-labeling was calculated for the identified lipids in the [U-^13^C]glucose samples. Since the major acyl chains found in yeast were 16:0, 16:1, 18:0, and 18:1 [31], the extracted ion chromatograms of glycerolipids of possible combinations of two acyl chains were prepared. In total, 2650 channels were generated for 60 molecular species (6 lipid class × 10 acyl chain combination) containing phosphatidylcholine (PC) [M+CH_3_COO]^-^, phosphatidylethanolamine (PE) [M-H]^-^, phosphatidylinositol (PI) [M-H]^-^, phosphatidylserine (PS) [M-H]^-^, phosphatidylglycerol (PG) [M-H]^-^, and diacylglycerol (DAG) [M+NH_4_]^+^ in 12 s. Of these, 16:0_16:1, 16:0_18:1, 16:1_16:1, and 16:1_18:1 were the major acyl chain combinations in all lipid classes except PG, which is a relatively minor glycerophospholipid in yeast [32] (Figure S4). One of the major yeast lipids PC 16:0_16:1 identified in unlabeled yeast accounted for 61.0%, 29.7%, 8.3%, and 1.6% of M+0, M+1, M+2, and M+3, respectively (Figure 4a,b). This highlights that isotope correction is also essential in lipid molecules measured in LC–MS. The natural isotopologue distribution was well-matched to the theoretical labeling calculated by isotope channel generator (Figure 4a), indicating that the isotopologue analysis was accurate. The effect of natural isotopes was corrected for 20 molecular species (five lipid class × four acyl chain combination) in 5 s using natural isotope subtractor. Figure 4c demonstrates the time-dependent increase in ^13^C-labeling in PC 16:0_16:1. The prominent isotopologues at 3 h were M+3, M+6 and M+8. These were presumed to be ^13^C-labeled molecules in the glycerol backbone and polar heads (Figure 4b). To compare the labeling dynamics depending on the difference in polar heads and acyl chains, fractional labeling was calculated within a second by using the fractional labeling calculator. Figure 4d–h clearly shows that labeling dynamics were different in the acyl chain combinations. The ^13^C-labeling dynamics of molecular species with double bonds in both acyl chains were commonly slower in all lipid classes than those with double bonds in only one of the acyl chains. These data suggest that the total number of double bonds regulates the turnover of acyl chains in glycerolipid and that the Isotope Calculation Gadget is able to capture the metabolic flow of lipids.

## 3. Materials and Methods

### 3.1. Software Development

The source codes were developed in Python ver. 3.7.6 operated on a Windows 10 Education 64 bit-based machine with an Intel (R) Core^TM^ i5-8350U CPU @ 1.70 GHz and 16 GB random access memory. Numpy ver.1.18.1, and tk ver 8.6.8 were used. All gadgets were operated in Garuda platform ver.1.4.

### 3.2. ^13^C-Tracing Culture of Cancer Cell Lines

HCT116 and WiDr cell lines were obtained from the Riken Bioresource Research Center (Tsukuba, Japan) and Japanese Collection of Research Bioresources Cell Bank (Osaka, Japan), respectively. [1-^13^C]glucose, [U-^13^C]glucose, and [U-^13^C]glutamine were obtained from Cambridge Isotope Laboratories (Andover, MA, USA, >99% purity). The culture conditions were as described previously except that the adhesion time was changed from 12 to 24 h [26]. Cells were collected at 24 h for the ^13^C-labeling analysis of intracellular amino acids and intermediates as described. 

### 3.3. ^13^C-Labeling Analysis of Water-Soluble Metabolites Using GC–MS

Intracellular metabolites of cancer cells were extracted using a methanol/water/chloroform method [26]. The dried metabolites were methoxyaminated by adding 50 µL of 40 mg/mL methoxyamine hydrochloride in pyridine and then *tert*-butyldimethylsilyated by adding 50 µL of *N*-(*tert*-butyldimethylsilyl)-*N*-methyltrifluoroacetamide containing 1% *tert*-butyldimethylchlorosilane (Thermo Fisher Scientific, Waltham, MA, USA) for GC–MS analysis, as described previously [27]. GC–MS analysis was performed using a GCMS-QP2020 (Shimadzu, Kyoto, Japan) equipped with a DB-5MS capillary column (Agilent Technologies, Santa Clara, CA, USA). GC–MS was operated under electron ionization at 70 electron volts. One microliter of the sample was injected at 250 °C using helium as the carrier gas at a flow rate of 1 mL/min. To analyze central metabolite derivatives, the GC oven temperature was held at 60 °C and increased to 325 °C at 10 °C /min for a total run time of approximately 30 min. The MS source and quadrupole were held at 230 °C and 150 °C, respectively. The ions were monitored in the SIM mode.

### 3.4. ^13^C-Tracing Culture of a Yeast Strain

The *Saccharomyces cerevisiae* BY4947 strain was obtained from National Bio-Resource Project (Japan). *S. cerevisiae* cells cultured on yeast extract peptone dextrose (YPD) plates containing 20 g/L bacto peptone, 20 g/L bacto yeast extract, 20 g/L glucose, and 20 g/L agar were inoculated into 5 mL of a YPD medium and cultured at 30 °C for 24 h with shaking at 150 rpm. The broth was transferred to 50 mL of a synthetic dextrose (SD) medium containing 20 g/L glucose and 6.7 g/L yeast nitrogen base without amino acids (Difco Laboratories, Detroit, MI, USA) in 200 mL flasks with an initial OD_600_ of 0.05. The pre-culture broth at 16 h was inoculated into the SD medium containing 5.0 g/L of [U-^13^C]glucose with an initial OD_600_ of 0.5 for main culture. The cells of the pre- and the main cultures were grown at 30 °C with shaking at 120 rpm. 

### 3.5. ^13^C-Labeling Analysis of Lipids Using LC-QTOF/MS

The yeast cells were collected with OD_600_ × volume (mL) = 12 and centrifuged at 9800× *g* for 10 min at 4 °C. The cell pellet was washed by 10 mL phosphate-buffered saline and centrifuged twice. Then, 400 µL of methanol was added to the pellet for quenching and stored at −80 °C. Cells were homogenized using a bead crusher (µT-12, TAITEC, Tokyo, Japan) at 3000 rpm for 3 min with glass beads (acid-washed 425–600 µm beads, Sigma Aldrich, St. Louis, MO, USA). Whole yeast lipids were extracted by a single-phase extraction method [29]. Then, 180 µL of the suspension was mixed with 20 µL of 4-fold-diluted EquiSplash (Avanti polar lipids, Alabaster, AL, USA) and 100 µL of chloroform in a new glass tube before being incubated for 2 h at room temperature. Then, 20 µL of water was added, and the samples were incubated for 10 min. Samples were centrifuged at 2000× *g* for 10 min, and the supernatants were transferred to analysis vials. The lipids were analyzed with a LC-QTOF–MS system (LCMS-9030, Shimadzu, Kyoto, Japan) using a previously described method [32], with some modification. Briefly, a 1 µL volume of sample was injected in to the ACQUITY UPLC Peptide BEH C18 (Waters). The flow-rate was 0.3 mL/min, and its temperature was set at 45 °C. The mobile phase (A) consisted of acetonitrile, methanol, and water (1:1:3, *v*/*v*/*v*). The mobile phase (B) was 2-propanol. EDTA and ammonium acetate were added to both (A) and (B) at final concentrations of 10 nM and 5 mM, respectively. The separation was performed using the following gradient: 0 min 0% (B), 1 min 0% (B), 5 min 40% (B), 7.5 min 64% (B), 12 min 64% (B), 12.5 min 82.5% (B), 18.5 min 85% (B), 19.5 min 95% (B), 19.6 min 0% (B), and 24.5 min 0% (B). MS analysis was performed using a data-dependent MS/MS acquisition (DDA) in positive (+) and negative (−) mode. The parameters were: MS1 and MS2 mass ranges, *m*/*z,* of 70–1750; MS1 event time of 250 ms; MS2 event time of 66 ms; number of MS2 events, 15; cycle time of 1240 ms; valid period of automatic exclude ions, 10 s; collision energy of 35 eV; collision energy spread of 20 eV; CID gas of Argon at 230 kPa; drying gas at 10.0 L/min (−) and 20.0 L/min (+); heating gas at 10.0 L/min; heat block temperature of 400 °C; DL temperatures of 300 °C(−) and 250 °C(+); interface temperatures of 300 °C(−), 100 °C(+); corona needle voltages of –3.5 kV(−), 4.5 kV(+); and nebulizer gas at 2.0 L/min(−) and 0.5 L/min(+). Data were analyzed with LabSolutions Insight (Shimadzu).

## 4. Conclusions

In this study, the open-source Isotope Calculation Gadgets, a software series that assists isotope-tracing experiments, was developed in a graphical user interface based-platform called Garuda. The software includes new features of method development, as well as conventional natural isotope correction, data interpretation, and mapping. Researchers can arrange the workflow according to their objectives to maintain smooth data transfer without manual processing, thus allowing isotope-tracing experiments to be flexible and easy-to-use. This software can contribute to the wide range of research and development in the field of metabolism.

## Data Availability

The data presented in this study are available in supplementary files. Source codes and blank metabolic maps can be found in https://github.com/NobuyukiOkahashi/Isotope_gadgets (accessed 1 June 2022).

## Supplementary Materials

The following supporting information can be downloaded at: https://www.mdpi.com/article/10.3390/metabo12070646/s1, File S1: Chemical_formula_SIM.csv; File S2: Example_data_SIM.csv. Figure S1. Labeling data visualization on a metabolic map ([1-13C]glucose and unlabeled glutamine); Figure S2. Labeling data visualization on a metabolic map ([U-13C]glucose and unlabeled glutamine); Figure S3. Labeling data visualization on a metabolic map (unlabeled glucose and [U-13C]glutamine); Figure S4. Heatmaps of glycerolipid abundance.
