# Supplementary material for: Isotope Calculation Gadgets: A Series of Software for Isotope-Tracing Experiments in Garuda Platform
Source: Metabolites. 2022 Jul 14;12(7):646. doi: 10.3390/metabo12070646 (PMC9318330; doi:10.3390/metabo12070646)
Supplement: Supplementary file 1 [file metabolites-12-00646-s001.zip › 220610_Supplementary figures.pdf]

Supplementary information

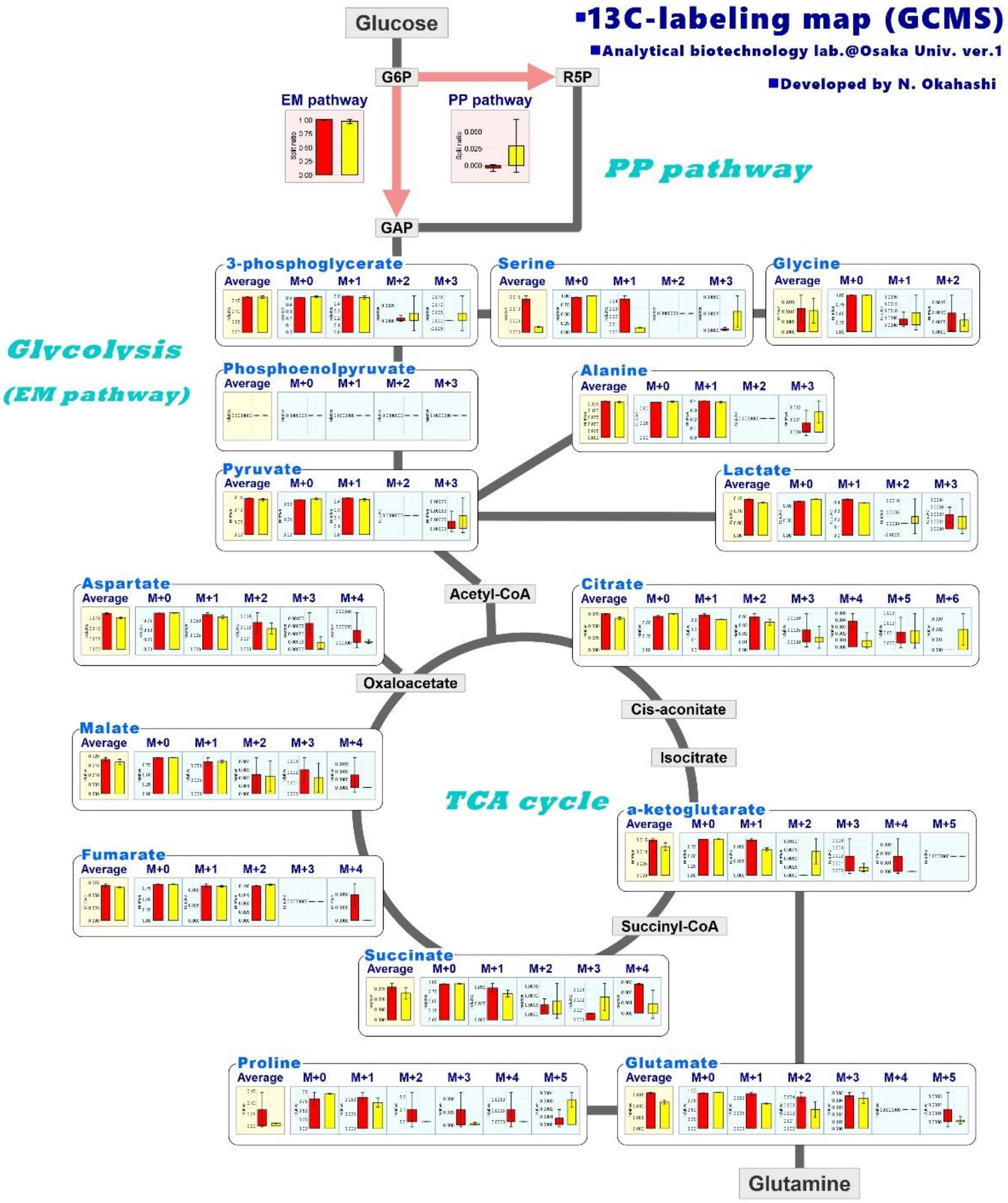

Figure S1. Labeling data visualization on a metabolic map ([1-^{13}C]glucose and unlabeled glutamine).

HCT116 (red) and WiDr (yellow) were cultured in medium containing [1-^{13}C]glucose and unlabeled glutamine. Blue, yellow, and pink boxes represent mass isotopologue distribution, fractional labeling, and split ratio, respectively.

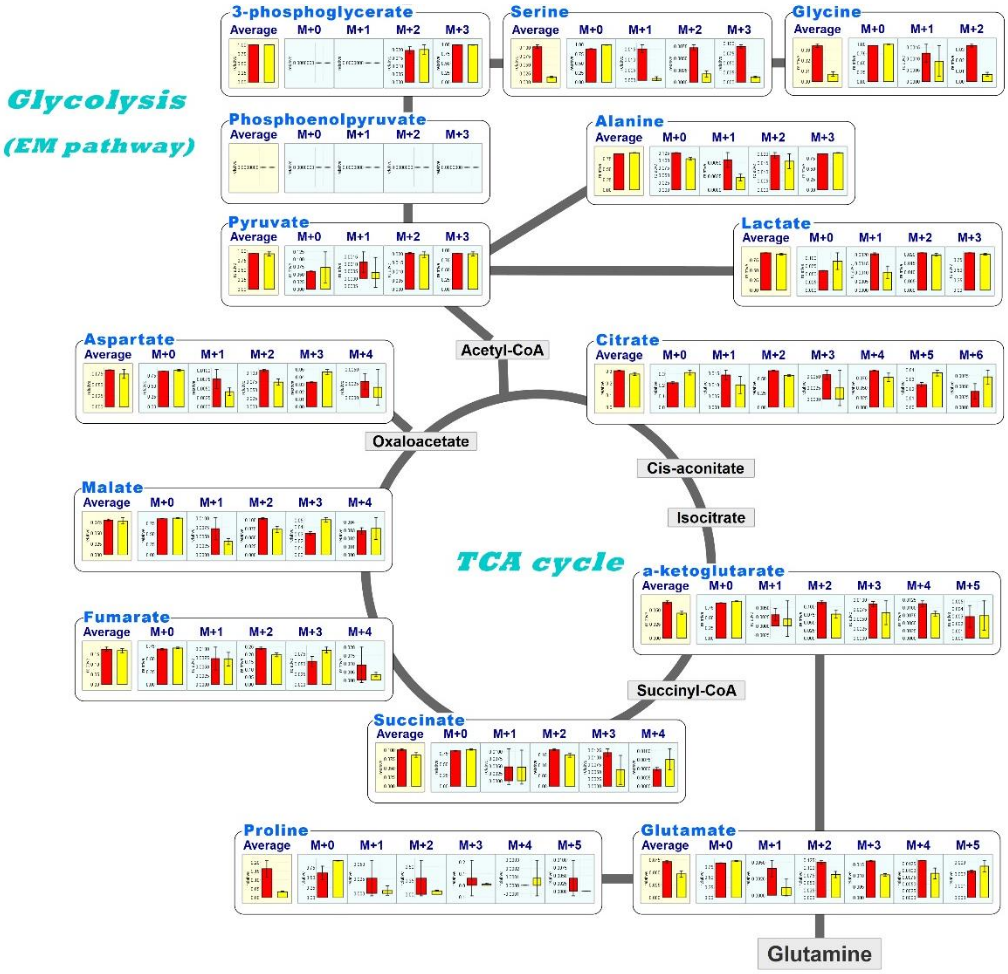

Figure S2. Labeling data visualization on a metabolic map ([U-^{13}C]glucose and unlabeled glutamine).

HCT116 (red) and WiDr (yellow) were cultured in medium containing [U-^{13}C]glucose and unlabeled glutamine. Blue and yellow boxes represent mass isotopologue distribution and fractional labeling, respectively.

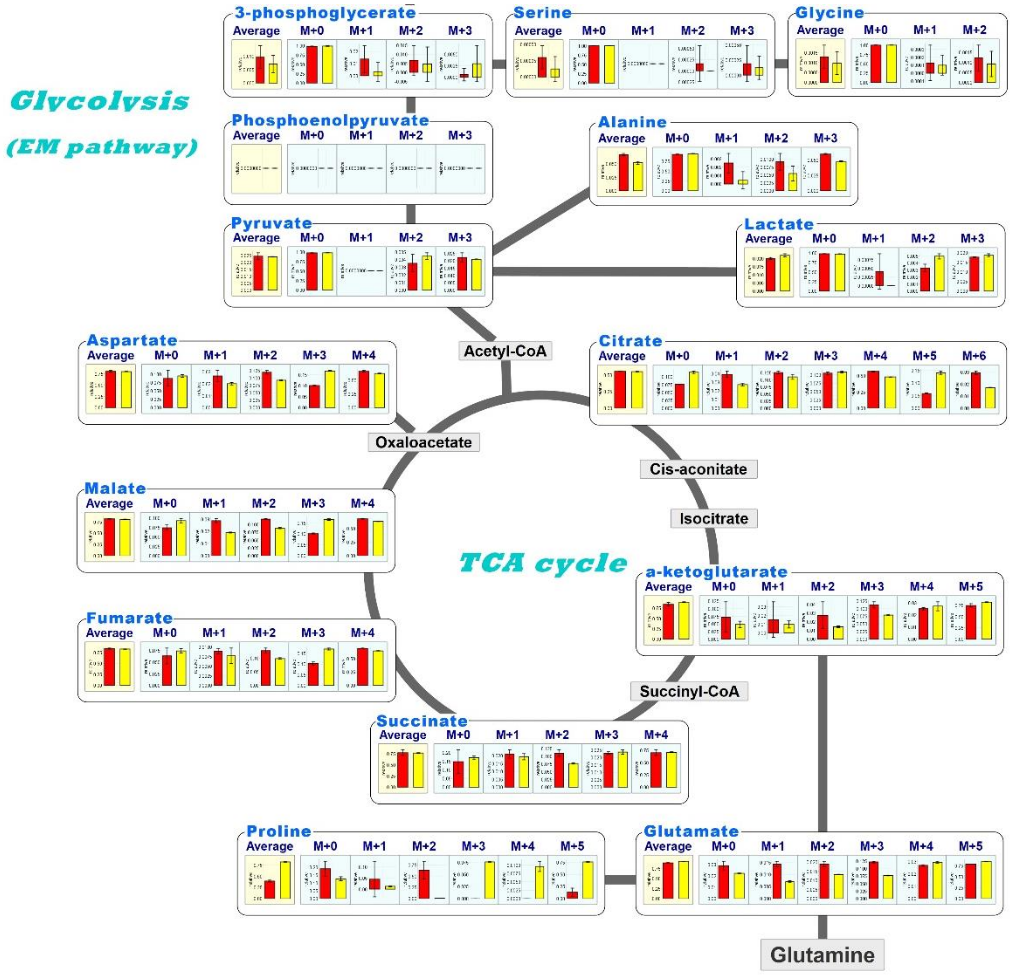

Figure S3. Labeling data visualization on a metabolic map (unlabeled glucose and [U-^{13}C]glutamine).

HCT116 (red) and WiDr (yellow) were cultured in medium containing unlabeled glucose and [U-^{13}C]glutamine. Blue and yellow boxes represent mass isotopologue distribution and fractional labeling, respectively.

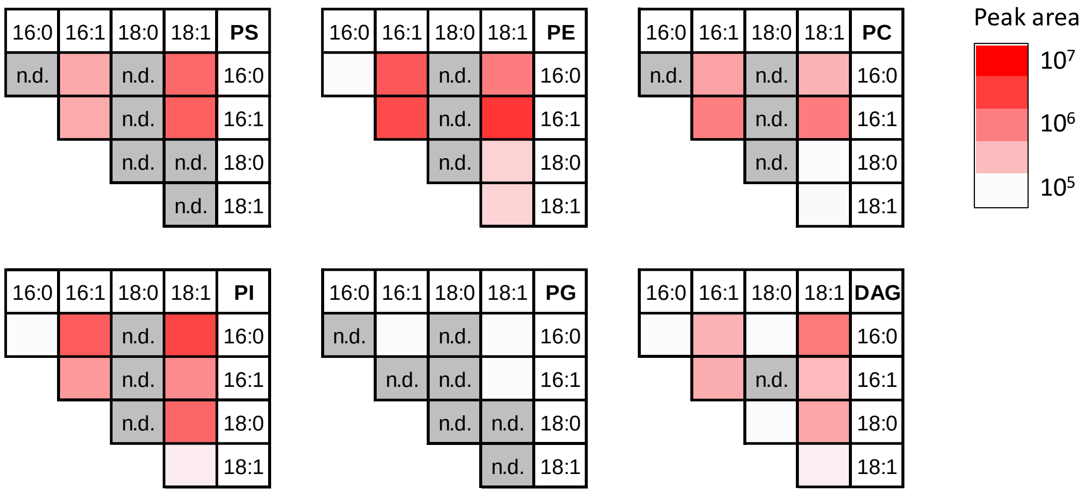

Figure S4. Heatmaps of glycerolipid abundance.

Peak areas of glycerolipids obtained from unlabeled yeasts were shown on the heatmaps. The columns and rows represent combinations of acyl chains.
